# Undernutrition and its association with socio-demographic, anemia and intestinal parasitic infection among pregnant women attending antenatal care at the University of Gondar Hospital, Northwest Ethiopia

**DOI:** 10.1186/s40748-018-0087-z

**Published:** 2018-09-12

**Authors:** Gemechu Kumera, Dereje Gedle, Animut Alebel, Fetuma Feyera, Setegn Eshetie

**Affiliations:** 1 Department of Public Health, College of Medicine and Health Science, Debre Markos University, Debre Markos, Ethiopia; 2 Department of Nursing, College of Medicine and Health Science, Debre Markos University, Debre Markos, Ethiopia; 3 School of Biomedical and Laboratory Sciences, College of Medicine and Health Science, University of Gondar, Gondar, Ethiopia

**Keywords:** Undernutrition, Pregnant women, Northwest Ethiopia

## Abstract

**Background:**

Under nutrition is a worldwide public health problem affecting the well-being of millions of pregnant women in the developing world. Only limited research has been conducted on the prevalence and determinants of maternal nutritional status in Ethiopia. Particularly, data on the nutritional status of pregnant women are lacking. The aim of this study was to assess the prevalence and determinants of undernutrition among pregnant women attending antenatal care at the University of Gondar Hospital, Northwest Ethiopia.

**Methods:**

An institution based cross-sectional study was conducted in January and February 2016. Randomly selected 409 pregnant women were included in the study. Nutritional status was estimated using mid-upper-arm circumference. Data on potential determinants of undernutrition were gathered using a structured questionnaire. The blood sample was collected to analyze hemoglobin. The stool sample was collected to identify intestinal parasitic infections. Statistical analysis was done using logistic regression. *P*-value < 0.05 at 95% confidence interval was considered as statistically significant.

**Results:**

The prevalence of undernutrition among pregnant women was 16.2% (95% CI: 12.4–20.1%). Using a logistic regression model, factors significantly associated with the undernutrition were living in rural areas (AOR = 2.26), low educational status [no formal education (AOR = 2.91), primary education (AOR = 2.69)], history of too many births (AOR = 2.55), anemia (AOR = 2.01), and intestinal parasitic infection (AOR = 2.73).

**Conclusion:**

The study findings provide evidence for the public health significance of under nutrition among pregnant women in the study area. The problem must be combated through rural livelihood promotion, socioeconomic empowerment of women, sustained nutrition education and expansion of family-planning services in the area.

## Background

Undernutrition and poor health from preventable causes disproportionately affect the well-being of millions of people in the developing world [1]. Maternal undernutrition is a worldwide public health problem affecting a high proportion of women in developing countries [2]. Women and young children are the most affected [3]. More than 3.5 million women and children under age five in developing countries die each year due to the underlying cause of undernutrition [4]. Women in sub-Saharan Africa, south-central and southeastern Asia are the most affected [3].

Ethiopia faces one of the world’s highest rates of maternal undernutrition. The analysis of Ethiopian Demographic Health Surveys (EDHS) 2000, 2005 and 2011 data revealed 30.5, 26.9, 27% of women in Ethiopia are undernourished respectively [5–7]. Based on recently conducted DHS country surveys, the proportion of undernourished women in sub-Saharan African countries ranges from 7 to 37%, indicating that maternal undernutrition in Ethiopia is higher than the level for many African countries [8].

Maternal prenatal undernutrition has a major impact on their own health as well as their children’s health. Increased perinatal and neonatal mortality, a higher risk of low birth weight and stunted babies, intrauterine growth restriction, stillbirths, and miscarriage are some of the consequences of undernutrition in women, which further undermines the human capital development of the family and society, and continues the cycle of poverty and malnutrition [9–15].

Undernutrition is a serious problem in Ethiopia. One of every four (27%) women in Ethiopia are undernourished [7]. Although the government and nongovernmental organizations have been perpetually working to improve the nutritional status of the women, the outcome of these efforts has not yet knocked mitigating effect on the prevalence of undernutrition among women in Ethiopia.

Only limited research has been conducted on the prevalence and determinants of maternal nutritional status in Ethiopia. Particularly, data on the nutritional status of pregnant women are lacking. Information on the nutritional status and associated factors among pregnant women are needed for prioritizing, designing and initiating intervention programs aimed at improving maternal nutrition. In addition, such data can be employed in programmes that are aimed to reduce maternal and child morbidity and mortality. Thus, this study was carried out to provide information regarding the prevalence and factors associated with undernutrition during pregnancy.

## Methods

### Study design, area and population

An institution based cross-sectional study was conducted at the University of Gondar Hospital in January and February 2016. The University of Gondar Hospital is a tertiary-level service-rendering institution that provides health service to over 4 million people in Gondar town and surrounding area. The Gondar town lies in the average at 2000 m above sea level and over 370,000 population reside in this administrative town [16]. The study populations were all pregnant women in Gondar town and surrounding area who attend antenatal care (ANC) at the University of Gondar Hospital.

### Sample size and sampling technique

A sample size of 409 was computed using single population proportion sample size calculation formula with inputs of 95% confidence level, 4% margin of error, 19.1% expected prevalence of undernutrition [17] and 10% non-response rate. A systematic random sampling technique was used to select the study subjects. According to the Hospital report, on average, 35–45 pregnant women visit the ANC daily, and 1241 pregnant women have been enrolled in ANC. Since the sample size was determined as 409, a sampling interval of 3 was used to select study participants. Of the first three pregnant women, one woman was randomly selected by using lottery method (The pregnant women is assigned to unique number (1, 2 and 3), putting it on a piece of paper. The pieces of paper are placed in the container and thoroughly mixed. Then, a blind folded researcher picks a number. Number three (3) was selected.). Accordingly, every 3rd pregnant women were selected to participate in the study until the required sample size of 409 pregnant women was obtained.

### Data collection methods

#### Questionnaire

A structured and pre-tested questionnaire was used for assessing potential determinants of undernutrition. The parts of the questionnaire on dietary diversity were adopted from Food and Nutrition Technical Assistance (FANTA) indicator guideline [18]. Other parts of the questionnaire were developed by the principal investigators. The questionnaire was administered in a local language(Amharic). The English version of the questionnaire was translated into local language and back to English by an expert to ensure its consistency. The translated Amharic version (local language) was pre-tested prior to the actual survey and modifications were made accordingly. The content validity of the tool was checked against the conceptual framework of the study by relevant professionals. Reliability of the tool was checked using a test-retest method. Questions with less than 0.7 kappa or Pearson coefficient values were removed or revised. Five data collectors (three midwives and two laboratory technicians) and one supervisor were recruited. Training was given for data collectors and supervisor by the principal investigator to have consensus and the same understanding of what is intended to be measured by each question in the questionnaire. The data collection process was followed daily by the supervisor and principal investigator. The dietary diversity (DD) level was assessed using 24-h recall method. Respondents were asked whether they had taken any food from predefined 12 food categories on a preceding day. Accordingly, the level of Dietary Diversity Score (DDS) was computed out of 12. According to the recommendation of Food and Agriculture Organization (FAO) of the United Nations, DD was classified into low (DDS ≤3), medium (DDS of 4 or 5), or high (DDS ≥6) [19]. Pregnant women from all trimesters (first, second and third) were included in the study.

#### Mid upper arm circumference (MUAC) measurement

Mid upper arm circumference was measured halfway between the tip of the shoulder (olecranon process) and the tip of the elbow (acromion process) to the nearest 0.1 cm. An insertion type MUAC tape that is non-elastic and non-stretchable was used to take the measurement. The measurement was taken at the mid-point on the relaxed left arm, without any clothing and with optimal tape tension (not too loose or not too tight) following the standard instructions and steps [20]. Recently published paper based on a review of evidence revealed that MUAC is a preferred anthropometric measurement during pregnancy [21] and also not affected by non-nutritional changes [22]. Undernutrition was defined as MUAC less than 22 cm [23].

### Laboratory analysis

#### Hemoglobin level determination

Venous blood was collected from each pregnant woman, using sterile blood collection tubes following standard procedures. Hemoglobin level was determined using hematological analyzer (Cell Dyn 1800, PD, USA) machine. Anemia was defined as a hemoglobin level of less than 11.0 g/dl during the first or third trimester or less than 10.5 g/dl during the second trimester [24]. According to the formula recommended by Center for Disease Prevention and Control, hemoglobin values were adjusted for altitude [25].

#### Stool specimen collection and examination

Stool samples were collected from participants using clean, dry and leak-proof cupped plastic container following standard procedures. The stool samples were masked, coded, and processed for parasitological examination. Different stool examinations were used for efficacy in detecting parasites. These were direct wet-mount and formaldehyde-ether sedimentation method [26, 27]. The WHO guide for diagnosis of intestinal parasitosis was used as an identification reference [28].

### Data processing and analysis

Data were entered using EPI-INFO version 7 software. The analysis was carried out using SPSS version 20 statistical program. Wealth index (poor, middle and rich) were computed using principal component analysis as a composite indicator of living standard. In this study, undernutrition (MUAC < 22 cm) was an outcome variable. Bivariate and multivariable logistic regression analysis was used to assess the association between the dependent and independent variables and to control confounders. Independent variables significantly associated with the dependent variable in bivariate regression models were exported to multivariable regression models for adjustment. The collinearity effect was tested using the Variance Inflation Factor (VIF) for all independent variables. Model fitness was assessed using the Hosmer-Lemeshow statistic test. *P*-value < 0.05 at 95% CI was considered statistically significant.

### Ethical cosideration

The study was conducted in confirmation of national and international ethical guidelines for biomedical research involving human subjects. Ethical clearance was obtained from an ethical review committee of the University of Gondar. Written informed consent was obtained from each study participants prior to participation in the study after the nature of the study was fully explained to the study participants. Women who had intestinal parasites and anemia were treated accordingly. Those women identified as undernourished were referred to ANC clinicians for treatment. Nutrition education was given to all study participants. Those participants identified as undernourished were given nutritional counseling and Ready Use Therapeutic Food (RUTF) in collaboration with the clinicians working in an ART clinic at Hospital.

## Results

### Socio-demographic characteristics of the study participants

Table 1 summarizes socio-demographic characteristics of the study participants. Of the total 409 pregnant women, initially planned for the study, 402 were volunteered to take part in the study, with a response rate of 98.3%. The vast majority of the respondents were Amhara in ethnicity (94.8%) and orthodox (88.6%) in religion. The majority of participants (62.4%) were in the age group 25–34 years with the mean and standard deviation of 26.6 (± 4.7) years. Nearly One-fourth of study subjects, 92(22.9%) had no formal education and more than half, 221 (55.0%) were housewives. The majority, 303 (75.4%), of the respondents, were urban dwellers. The average household size was 3.2 (± 1.6). The median monthly household income was 2000 Ethiopian birr.

**Table 1:** Socio-demographic characteristics of the study participants, Northwest Ethiopia, 2016 (*n* = 402)

Characteristics		Frequency (n)	Percent (%)
Age(years)	15–24	120	29.9
	25–34	251	62.4
	≥ 35	31	7.7
Residence	Urban	303	75.4
	Rural	99	24.6
Marital status	Married	386	96
	Single	7	1.7
	Divorced	2	0.5
	Separated	6	1.5
	Widowed	1	0.2
Religion	Orthodox	356	88.6
	Muslim	37	9.2
	Protestant	9	2.2
Ethnicity	Amhara	381	94.8
	Tigre	15	3.7
	Oromo	2	0.5
	Other	4	1.0
Educational status	No formal education	92	22.9
	Primary education	84	20.9
	High school education	112	27.9
	Certificate and above	114	28.4
Occupation	House wife	221	55.0
	Farmer	13	3.2
	Merchant	51	12.7
	Government employee	94	23.4
	Daily laborer	11	2.7
	Student	12	3.0
Family size	≤ 3	285	70.9
	4–6	90	22.4
	> 6	27	6.7
Wealth Index	Low	134	33.3
	Middle	134	33.3
	High	134	33.3

### Environmental and sanitation factors

Table 2 summarizes environmental and sanitation characteristics of the study participants. Source of drinking water for the greater number, 368(91.5%) of study subjects was tap water. The majority of study participants, 383(95.3%) had toilet facilities. The large proportion of participants, 283(73.9%) use pit latrine.

**Table 2:** Environmental and sanitation characteristics of pregnant women attending antenatal care, Northwest Ethiopia, 2016

Characteristics		Frequency (n)	Percent (%)
Source of drinking water	Tab	368	91.5
	Well	11	2.7
	Spring	23	5.7
Possession of toilet facility	Yes	383	95.3
	No	19	4.7
Types of latrine	Pit latrine	283	73.9
	Water flush	97	25.3
	Public	3	0.8

### Reproductive health factors

Table 3 summarizes reproductive health factors of study participants. More than half, 224 (55.7%) of the participants were in the third trimester, 117(29.1%) in the second and 61(15.2%) in the first trimester. The results revealed a greater majority of the participants, 198(49.3%) had a first pregnancy at a time of data collection. In 33 (8.2%) of the study participants, the birth interval was less than the recommended 24 months. The mean parity was 1.1 (± 1.6), ranging between 0 and 7 pregnancies. The mean MUAC for nulliparas was 24.7 (+/− 2.6) cm. The corresponding value for parity categories of 1–3 and 4 or more was 25.2 (+/− 3.1) cm and 23.5 (+/− 2.8) cm, respectively.

**Table 3:** Reproductive health factors among pregnant women attending antenatal care, Northwest Ethiopia, 2016

Characteristics		Frequency (n)	Percent (%)
Trimester of pregnancy	First	61	15.2
	Second	117	29.1
	Third	224	55.7
Parity	0	198	49.3
	1–3	155	38.6
	≥ 4	49	12.2
Birth interval	0(No birth)	198	49.3
	< 2 yr	33	8.2
	≥ 2 yr	171	42.5

### Dietary intakes of the study participants

**Fig. 1:**
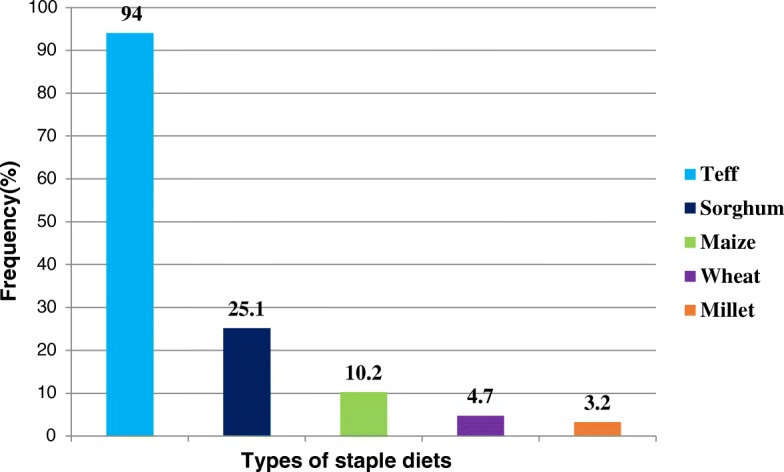
Staple diets of pregnant women attending antenatal care, Northwest Ethiopia, 2016

Table 4 summarizes dietary intakes of the study participants. The vast majority of the study subjects consumed cereal-based foods (made of teff) (Fig. 1). The commonly and frequently consumed foods were a starchy staple, 100% and legumes, 273(67.9%). Only one-fourth, 86(21.4%) of study subjects were consumed vitamin A rich fruits and vegetables in the reference period. Two hundred forty-six, (61.2%) of the study participants reported that they consumed coffee. A small proportion, 128 (31.8%) of study participants reported that they consumed a diet of animal origin prior to the survey. Among animal products, flesh meat was consumed by 94 (23.4%) of the study subjects, whereas egg, milk and milk products and organ meat were consumed by 20 (5%), 25 (6.2%) and 2 (0.5%), respectively. The mean DDS was 3.33 (± 0.77), ranging between 1 and 7. The majority of study participants, 267 (66.4%) had low DDS (≤3 food groups). The meal frequency was three times a day for the majority of the study participants 282(70.1%).

**Table 4:** Dietary intakes of pregnant women attending antenatal care, Northwest Ethiopia, 2016

Characteristics		Frequency (n)	Percent (%)
Number of meals/day	**<** 3	46	11.4
	3	282	70.1
	**>** 3	74	18.4
Family food source	Grow their own	70	17.4
	Buy/purchase	330	82.1
	Subsidies/food aid	2	0.5
Dietary diversity	Low	267	66.4
	Medium	123	30.6
	High	12	3.0
Home gardening	Yes	31	7.7
	No	371	92.3
Animal food source	Yes	128	31.8
	No	274	68.2
Coffee intake	Yes	246	61.2
	No	156	38.8
Number of cup of coffee per day	≤ 3	190	77.2
	> 3	56	22.8

### Anemia, HIV and diarrhea among the study participants

Of all study participants, 17(4.2%) were positive for HIV. Among 21 study participants who were positive for HIV, 6(28.6%) were undernourished. The mean MUAC for HIV positive and negative were 23.1(± 3.8) and 24.8(± 2.8) cm, respectively. A small proportion, 26(6.5%) of the pregnant women had diarrhea; of whom nearly one-fourth, 6(23.1%) were undernourished. Overall, the prevalence of anemia was 132(32.8%), and was more evident in undernourished pregnant women (25%) than in normal pregnant women(MUAC≥ 22 cm).

### Prevalence and type of intestinal parasites among the study participants

**Fig. 2:**
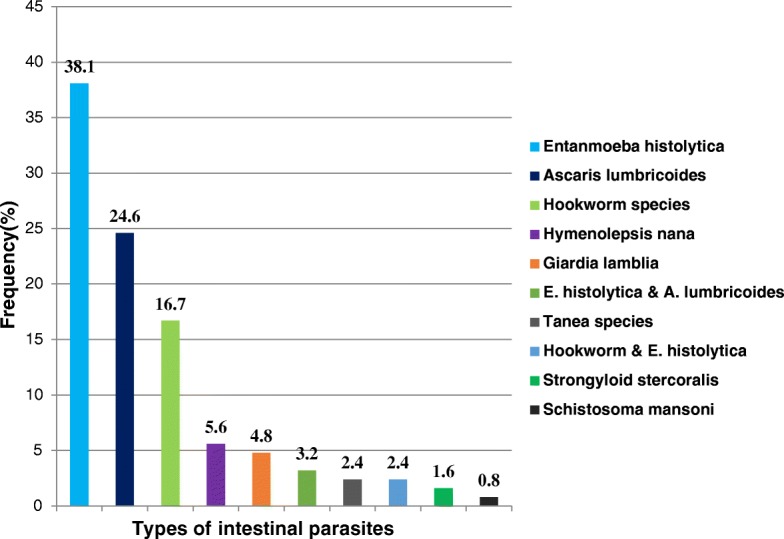
Prevalence and types of intestinal parasites among pregnant women attending antenatal care, Northwest Ethiopia, 2016

Figure 2 summarizes prevalence and type of intestinal parasitic infection among the study participants. One hundred twenty-six, (31.3%) of study participants were infected with one or more intestinal parasites. Of whom, 35(27.8%) were undernourished. The most common single and mixed parasites observed were *Entamoeba histolytica* 48(38.1%) and Ascaris lumbricoides 31(24.6%). Intestinal parasitic infection tends increase among rural dwellers and pregnant women who used other sources of drinking water other than tap water. Of 99 rural dwellers pregnant women, 41(41.4%) were infected with one or more intestinal parasites. Among 11 pregnant women who consumed well water, 7(63.6%) were infected with one or more intestinal parasites. Similarly, among 23 pregnant women who consumed spring water, 12(52.2%) were infected with one or more intestinal parasites.

### Prevalence of under nutrition

Undernutrition (MUAC < 22 cm) was observed in 65 of 402 pregnant women studied, yielding a prevalence of 16.2% (95% CI: 12.4–20.1%). The mean MUAC (±SD) of the study participants was 24.8 cm (± 2.9). The value ranged from 18 to 37 cm. The prevalence of undernutrition during the first, second and third trimesters were, 14.8, 16.2 and 16.5%, respectively. The mean MUAC(±SD) for the first, second and third trimesters were, 24.8 (± 3.2), 24.8(± 2.8) and 24.7 (± 2.7) g/dl, respectively.

### Factors associated with undernutrition

Table 5 summarizes factors associated with undernutrition among pregnant women. A multivariable analysis in a form of logistic regression was employed to identify the risk factors of undernutrition among pregnant women. The analyses rest on two outcomes of nutritional status of pregnant women: whether they are undernourished (MUAC< 22 cm) or not (MUAC≥ 22 cm). In the bivariate analysis, undernutrition was significantly associated with age, residence, wealth index, educational status, source of drinking water, parity, animal source food, HIV, anemia and intestinal parasite. The multivariable logistic regression analysis revealed that residence, maternal educational status, parity, anemia and intestinal parasite were predictors of undernutrition.

**Table 5:** Factors associated with the undernutrition among pregnant women

Predictors	Under nutrition		COR (95%CI)	AOR (95% CI)	*P*- values
	Yes	No			
Age					
15–24	34	86	2.36(1.01, 5.53)		
25–34	28	223	2.17 (0.87, 5.44)		
≥ 35	3	28	1		
Residence					
Rural	32	67	3.91(2.24, 6.81)	2.26 (1.06, 4.81)	0.03*
Urban	33	270	1	1	
Wealth index					
Low	36	98	4.54 (2.13, 9.70)		
Middle	19	115	2.05(1.12, 3.75)		
High	11	123	1		
Educational status					
No formal education	31	61	5.18(2.37, 11.31)	2.91 (1.11, 7.56)	0.02*
Primary education	12	72	4.32(2.06, 9.03)	2.69 (1.09, 6.64)	0.03*
Secondary education	10	102	3.04(1.44, 6.44)		
Certificate and above	12	102	1	1	
Source of drinking water					
Spring	8	15	2.54 (1.00, 6.47)		
Well	3	8	2.16 (0.86, 5.48)		
Tab	54	314	1		
Parity					
No birth	23	175	1		
1–3	24	131	3.63 (1.76, 7.51)		
≥ 4	19	30	4.82 (2.34, 9.91)	2.55 (1.04, 6.26)	0.04*
Animal source food					
Yes	13	115	1		
No	52	222	2.07 (1.08, 3.96)		
HIV					
No	60	325	1		
Yes	5	12	2.38 (1.01, 5.95)		
Anemia					
No	32	238	1	1	
Yes	33	99	2.48 (1.45, 4.25)	2.01 (1.16, 3.79)	0.01**
Intestinal parasite					
Absent	30	246	1	1	
Present	35	91	3.15 (1.83, 5.43)	2.73 (1.48, 5.03)	0.001**

^*^P-value is significant at < 0.05, ^**^P-value is significant at ≤ 0.01

Place of residence was significantly associated with nutritional status of pregnant women (*P* = 0.03). The risk of undernutrition was two times, [AOR = 2.26; 95% CI (1.06, 4.81)] higher among rural pregnant women than their urban counterparts.

The study also witnessed significant association between educational level and maternal nutritional status. The risk of undernutrition for pregnant women with no formal education and primary education was 2.91 and 2.69 times higher, respectively as compared to pregnant women with higher education (certificate and above) [AOR = 2.91; 95% CI (1.11, 7.56)], [AOR = 2.69; 95% CI (1.09, 6.64)].

Significant associations were also observed between nutritional status and parity. The risk of undernutrition for pregnant women with grand multiparas was two and half times higher as compared to pregnant women with no parity [AOR = 2.55; 95% (1.04, 6.26)].

Anemia was found to be significantly associated with undernutrition. Pregnant women who were anemic were two times more likely to be undernourished than those with normal hemoglobin level [AOR = 2.01; 95% (1.16, 3.79)].

The intestinal parasite was also found to be significantly associated with undernutrition. Pregnant women who had one or more intestinal parasitic infection were three times more likely to be malnourished as compared to those who had no intestinal parasitic infection [AOR = 2.73; 95% CI (1.48, 5.03)].

In this study, nutritional status did not show any statistical association with regard to dietary diversity, family size, occupation, latrine availability, number of meals per day, coffee intake, staple diets, home gardening and family food source.

## Discussion

This study assessed the prevalence and determinants of undernutrition among pregnant women attending antenatal care. The current study witnessed the public health significance of undernutrition in the study area. Findings of the study showed that, 16.2% of pregnant women were undernourished. In Ethiopia, few studies determined the prevalence of undernutrition in pregnant women and came up with figures 9.2% [29], 19.1% [17], 31.4% [30] and 34.0% [31]. The studies consistently witnessed the public health significance of undernutrition in the country. The current study showed that prevalence of undernutrition among pregnant in the country is decreasing as compared to earlier studies. The probable reason for this variation could be the interventions on nutrition, maternal health and other women empowering programs by the government and other non-governmental organizations in the country. Moreover, the variation may be due to geographical variation, smaller land holding, the recurrent food insecurity and the seasonal difference in data collection. Further, the prevalence may have overestimated in earlier studies as only adolescent pregnant women were included in the study.

Women living in rural areas are more undernourished than their urban counterparts. Similar results have been reported from earlier studies [3, 6, 32, 33]. The disparity may be due to less developed infrastructures, low nutritional awareness, low access to health care, safe water, and sanitation facilities, traditional ways of farming as only means of surviving and cultural and religious influences in rural areas. In addition, lower nutritional status among rural pregnant women could be explained partly by the fact that in rural areas women are more vulnerable to early marriage and childbearing than women in urban areas [6]. Hence, in addition to their own health needs, they need an adequate dietary intake for pregnancy and child growth. Further, the higher levels of labor or workload among rural pregnant women may contribute to poor nutritional status among pregnant women in rural areas, as overexertion is a predisposing factor to maternal nutritional depletion [34].

The study findings indicate a negative relationship between the educational status of the pregnant women and undernutrition, with decreasing educational levels from certificate and above to no formal education, the level of undernutrition tends to increase. Previous studies also have documented similar findings [30–32, 35]. The probable reason could be pregnant women who are literate may have more exposure to media, which influence their behaviour in matters related to their own feeding and health [36]. Unawareness of illiterate women about their own health and nutritional status could be another reason associated with their poor nutritional status. Moreover, the low educational level of women could be associated with little or no decision-making power in the household about food distribution, purchase of household consumption items and financial issues which may contribute to their low nutritional status. However, in a study conducted in Iran, there was no statistically significant association between pregnant women’s education level and nutritional status [37]. The variation can be due to the difference in socioeconomic status and nutritional care prior to and during pregnancy.

Another finding that affects pregnant women’s nutritional status is parity. The odds of undernutrition were observed to rise as parity advances. Previous studies also reported the same [29, 31, 32]. The study witnessed the deleterious effect of too many births on nutritional status. The finding is consistent with the knowledge that repeated reproductive cycles deplete maternal nutrition store. Moreover, higher-level parity obligates women to take care of their children rather than protecting their own health and nutritional status, given limited household resources.

The current study finding witnessed a significant positive association between anemia and undernutrition. The proportion of undernutrition was significantly more among anemic pregnant women compared to normal hemoglobin level pregnant women. This finding is in line with studies conducted in Ethiopia [38], Kenya [39] and India [40], which indicated that the risk of anemia tends to increase among undernourished pregnant women. This might be due to the fact that undernourished pregnant women have a higher risk of being deficient in micronutrients and therefore more likely to be anemic. As the study is cross-sectional, it is not viable to exclude “the chicken or the egg” causality dilemma. However, as protein is known to take part in multiple metabolic pathways, it might have a causal role in anemia. This relation between undernutrition and anemia might be due alterations in spleen and bone marrow erythropoiesis, reduction in reticulocyte as a result of a protein-energy deficiency in undernourished pregnant women [41].

In the present study, intestinal parasitic infection was found to be one of the determinant predictors of risk of undernutrition. Pregnant women who had one or more intestinal parasitic infection were at greater risk of being undernourished as compared to pregnant women with no intestinal parasites. Intestinal parasitic infections may affect nutritional status by reducing appetite and dietary intake. It also affects by increasing nutrient losses due to vomiting, diarrhea, poor digestion and absorption. Further, the nutrient demand of parasite itself and intestinal bleeding caused by the parasite may contribute to poor nutritional status [42–44].

Major strength of this study was the random selection of the study participants. The major limitation was the cross-sectional nature of its design as we can’t establish causal relationships between the independent variables and nutritional status of the pregnant women. Secondly, the study was institution based and the study subjects may not represent the general population. Third, the last date of the menstrual period and/or fundal height with a subsequent urine test, were used to diagnose pregnancy, which may not accurately confirm pregnancy**.**

### Recommendation

A national level study should be conducted in Ethiopia. The problem must be combated through an implementation of strategies like rural livelihood promotion, socio-economic empowerment of women and expansion of women’s education, particularly in rural areas. Nutritional care should be integrated into maternity services. We also suggest sustained nutrition education to enhance good nutritional awareness and practice of pregnant women. Expansion of family planning services will also have affirmative input. Prevention and control of intestinal parasite infection through improving access to safe and adequate water supply; sanitation and hygiene practices should be considered. Screening of pregnant women for intestinal parasitic infections, universal deworming programs, and expansion of prenatal iron-folate supplementation should also be looked for.

## Conclusion

The study findings provide evidence for the public health significance of undernutrition among pregnant in the study area. The important risk factors/ predictors of undernutrition (MUAC < 22 cm) were living in rural areas, low educational status, history of too many births, anemia and intestinal parasitic infection.

## Abbreviations

ANC: Antenatal care
DD: Dietary diversity
DDS: Dietary diversity score
EDHS: Ethiopian Demographic and Health Survey
FANTA: Food and Nutrition Technical Assistance
FAO: Food and Agriculture Organization of the United Nations
MUAC: Mid upper arm circumference
SD: Standard deviation
